# Measuring individual differences in cognitive abilities in the lab and on the web

**DOI:** 10.1371/journal.pone.0226217

**Published:** 2019-12-11

**Authors:** Simón Ruiz, Xiaobin Chen, Patrick Rebuschat, Detmar Meurers

**Affiliations:** 1 LEAD Graduate School and Research Network, University of Tübingen, Tübingen, Germany; 2 Department of Linguistics and English Language, Lancaster University, Lancaster, United Kingdom; 3 Department of Linguistics, University of Tübingen, Tübingen, Germany; Universidad de Chile, CHILE

## Abstract

The present study compared lab-based and web-based versions of cognitive individual difference measures widely used in second language research (working memory and declarative memory). Our objective was to validate web-based versions of these tests for future research and to make these measures available for the wider second language research community, thus contributing to the study of individual differences in language learning. The establishment of measurement equivalence of the two administration modes is important because web-based testing allows researchers to address methodological challenges such as restricted population sampling, low statistical power, and small sample sizes. Our results indicate that the lab-based and web-based versions of the tests were equivalent, i.e., scores of the two test modes correlated. The strength of the relationships, however, varied as a function of the kind of measure, with equivalence appearing to be stronger in both the working memory and the verbal declarative memory tests, and less so in the nonverbal declarative memory test. Overall, the study provides evidence that web-based testing of cognitive abilities can produce similar performance scores as in the lab.

## Introduction

Individual differences can greatly affect how we acquire and process language [1–3] and mediate and/moderate the effectiveness of instruction [4]. In adult language learning, for example, learners’ cognitive abilities have great explanatory power in accounting for differences in learning outcomes ([5–6]). For instance, working memory and declarative memory are considered to be particularly important sources of learner variation (e.g., [7–10]; see [4, 11], for reviews).

The effect of working memory and declarative memory on language learning has been primarily studied in lab settings, i.e., in well-controlled environments where participants are tested individually. While this choice is methodologically sound, it can also negatively affect sample size and population sampling [12, 13, 14]. Lab-based testing generally means testing participants individually and sequentially, which is labor-intensive and could explain why lab studies tend to have (too) few participants to allow for meaningful generalization. As an example, in second language (L2) research, Plonsky [13] found that the typical sample size in L2 studies was 19 participants, and Lindstromberg [15] recently reported a similar small average sample size of 20 participants. In the same vein, [16] reported that, in psychology, median sample sizes have not increased considerably in the last two decades, and are generally too small to detect small effect sizes, which are distinctive of many psychological effects. Moreover, many (if not most) lab studies in research draw their sample from the surrounding student population, which is understandable given the ease of access, but also means that samples are often not representative of the population of interest. Conducting research by means of remote testing via the web could alleviate some of these concerns. For example, web-based testing facilitates the acquisition of large amounts of data since participants can be tested simultaneously, enabling researchers to run higher-powered studies. Likewise, test administration can also be more cost-effective than research conducted in the lab [17].

The use of (remote) web-based testing can also offer other important methodological advantages over other forms of simultaneous delivery of tests, such as traditional paper-pencil and (offline) computer-based testing [18, 19]. Particularly, it allows researchers to standardize and optimize testing procedures, which can contribute to more consistent and uniform test-taking conditions across different locations and times [20]. This, in turn, can also facilitate the replication of studies [21]. Moreover, remote testing via the web can reduce experimenter effects, as testing can occur in more ecologically-valid settings, and without any direct contact between experimenters and participants [20, 21]. Finally, and more importantly, web-based experimenting has been found to be a reliable and effective research tool [17, 22, 23].

The present study compared lab-based and web-based versions of cognitive tests that are widely used in disciplines such as psychology and second language research. Particularly, our intent was to compare performance of measures as they are originally used in the lab with their corresponding online versions. In doing so, our objective was to validate the web-based tests for use in subsequent research and to make these available to the wider research community, and especially to researchers working on the area of L2 acquisition. The sharing of tasks, especially of tasks that permit the collection of substantial amounts of data via the web, will be an important component in alleviating the data collection issues associated with lab-based research. Moreover, making these specific tasks available will also contribute directly to our understanding of individual differences in L2 acquisition. To support such task sharing and use, it is essential to first establish the validity of the online versions of the tasks (on a par with what is established about the offline versions). With this in mind, the study set out to establish measurement equivalence between lab-based and web-based tests of working memory and declarative memory.

According to Gwaltney, Shields and Shiffman ([24], p. 323), measurement equivalence can be established if “1) the rank orders of scores of individuals tested in alternative modes closely approximate each other; and 2) the means, dispersions, and shapes of the score distributions are approximately the same”. The first type of equivalence regards to whether differences observed in one measurement are also systematically found in the other, meaning that, even when the two measurements produce two different numbers, these numbers are clearly and systematically associated with each other. The second type concerns whether two measurements yield the same numbers. Considering that this study is a subcomponent of the dissertation research of the first author, limiting funding and time (see limitations below), we focused the investigation on one type of measurement equivalence, the first type: Do people who have relatively high values in one of tests also have relatively high values on the other test, and the other way around? More specifically, we compare the differential performance generated by two versions of tests measuring working memory and declarative memory abilities in lab-based and web-based settings, in order to assess whether the two versions are equivalent regarding the relationships between scores.

Assessing equivalence between lab and web-based measurements is essential for several reasons. Firstly, it is necessary to demonstrate that the findings obtained in web-based studies are comparable to those of previous research, which have been mainly collected in lab-based settings. Secondly, it is important to ensure that cognitive constructs are similarly gauged in both testing modalities. Likewise, it is crucial to establish whether lab-based and web-based tests are equivalent, given that web-based testing could prove to be a viable way to tackle some of the current methodological issues found in research conducted in lab-based settings, such as underpowered studies, restricted population sampling, and small sample sizes [17, 22, 23]. Of these methodological issues, in particular, low statistical power and small sample sizes have been identified as key factors in the ongoing discussions about the reproducibility of research findings in life and social sciences [25–27]. In psychology, for example, there is currently considerable debate about the so-called *replication crisis* [28], that is, failure to reproduce significant findings when replicating previous research [27]. In this regard, and considering that much research is underpowered [29, 30], web-based testing can enable the collection of larger sample sizes, and thus contribute to achieve more statistical power to detect the effects of interest. On the other hand, the ease of access, cost-effectiveness, and practicality of web-testing can also increase the attempts to reproduce results from previous studies, and thus making (large-scale) replication studies more appealing for researchers to undertake [30].

### Working memory

Working memory is the capacity to process and hold information at the same time while performing complex cognitive tasks such as language learning, comprehension and production [31]. According to Baddeley and colleagues (e.g., [32]), working memory is a multicomponent system that includes storage subsystems responsible for retaining both visual-spatial and auditory information, an episodic buffer that serves as a link between the storage subsystems and long-term memory, and a central executive that acts as an attentional control system.

Regarding L2 learning, working memory assists learners to simultaneously process form, meaning and use of language forms. More specifically, working memory is involved in key cognitive processes such as decision making, attention control, explicit deduction, information retrieval and analogical reasoning [4]. Moreover, working memory is also important for retaining metalinguistic information while comprehending and producing L2 language [33]. In this regard, meta-analytic work has reported the important role of working memory in L2 comprehension and production (e.g., [34–36]). For example, Linck et al. ([36], p. 873) found that working memory has a positive impact on L2 comprehension outcomes (*r* = .24). Likewise, Jeon and Yamashita’s [35] meta-analysis also showed that working memory is related to L2 reading comprehension (*r* = .42). Regarding production, meta-analytic research has, too, indicated a significant association with working memory (e.g., [36]). In this case, Linck et al. ([36], p. 873) found a positive correlation for productive outcomes as well (*r* = .27).

Working memory is often measured by means of simple or complex span tasks. Simple span tasks, such as digit span and letter span, entails recalling short lists of items, and they seek to measure the storage component of working memory [37]. Complex span tasks, such as the operation span task (OSpan; [38]), on the other hand, include remembering stimuli while performing another task. This type of tasks taxes both processing (attention) and storage (memory) aspects of working memory [32]. Here, we focus on a complex task, namely the OSpan. This complex task has been found to be a valid and reliable measure of working memory capacity [39], and has also been recommended as a more accurate measure to examine the association between working memory and L2 processing and learning [40].

### Declarative memory

Declarative memory is the capacity to consciously recall and use information [41]. The declarative memory system is one of the long-term memory systems in the brain [42]. It is mainly responsible for the processing, storage, and retrieval of information about facts (semantic knowledge) and events (episodic knowledge; [43, 44]). Learning in the declarative memory system is quick, intentional, and attention-driven [45].

Substantial research has now investigated the role of declarative memory in first and second language acquisition [46]. In first language acquisition, declarative memory is involved in the processing, storage and learning of both arbitrary linguistic knowledge (e.g., word meanings) as well as rule-governed aspects of language (e.g., generalizing grammar rules [47, 48]). In the case of L2 acquisition, declarative memory underpins the learning, storage and processing of L2 vocabulary and grammar [47, 48], at least in the earliest phases of acquisition [46, 49]. Several studies (e.g., [2, 9, 49, 50]) has confirmed the predictive ability of declarative memory to explain variation in L2 attainment.

Declarative memory has been tested through recall and recognition tasks (e.g., 49, 50), both verbal, such as the paired associates subtest of the Modern Language Aptitude Test (MLAT5; [51]), and nonverbal, such as the Continuous Visual Memory Task (CVMT; [52]).

### The present study

The main goal of the present study was to provide web-based versions of commonly employed individual difference measures in second language research, in order to make them usable in large-scale intervention studies (generally in authentic, real-life learning contexts). To that end, we examined whether lab-based and web-based versions of working memory and declarative memory tests yield similar performance scores, i.e., whether the two versions were equivalent or comparable. More specifically, we assessed whether the values of one type of mode of administration corresponded to the values in the other mode (i.e., first type of equivalence). In other words, are the differences in scores constant, or parallel in the two ways of measuring? The web-based versions are freely available; to use the test, please send an email to the first author.

## Methods

### Ethics statement

This research was approved by the Commission for Ethics in Psychological Research, University of Tübingen, and all participants provided written informed consent prior to commencement of the study.

### Participants

Fifty participants (37 women and 13 men), with a mean age of 26.4 years (*SD* = 4.2), partook in the study. The majority of participants were native speakers of German (72%), followed by Russian (8%), Spanish (6%), Chinese (4%), English, Hungarian, Persian, Serbian and Vietnamese (2% each). Seven (14%) participants did not complete the second half of the study (i.e., web-based testing). Additionally, participant numbers differed across test versions due to technical difficulties (i.e., participants entered their responses using the wrong keys [Web-based CVMT]; and data was not correctly saved for one participant [Web-based MLAT5]; see description and Table 1 below, and Discussion). Twenty-seven participants were graduate students (54%), and twenty-three were undergraduates (46%). Participants self-reported English proficiency, with most being advanced learners (82%), followed by intermediate (18%). All subjects gave informed consent and received €20 for participating.

**Table 1:** Descriptive statistics for comparison of lab-based and web-based testing.

Test	N	*M*	*SD*	Skew	Kurtosis
OSpan Lab-based	50	25.78	13.34	0.61	2.90
OSpan Web-based	43	29.79	15.42	0.67	3.26
MLAT5 Lab-based	50	17.92	5.50	-0.64	2.49
MLAT5 Web-based	42	19.10	5.81	-1.19	3.58
CVMT Lab-based	49	1.99	0.46	0.23	3.35
CVMT Web-based	40	2.30	0.63	0.73	3.32

Note: OSpan = Automated Operation Span Task; Verbal declarative memory test: MLAT5 = Modern Language Aptitude Test, Part 5; Nonverbal declarative memory test: CVMT = Continuous Visual Memory Task.

### Materials

Three cognitive tests were administered, one measuring working memory capacity, and two assessing verbal and nonverbal declarative memory abilities, respectively. In the lab-based setting, both working memory and nonverbal declarative memory tests were programmed and delivered via E-Prime v2.0 [53]; the verbal declarative memory test was given in paper-pencil form, as originally developed and delivered. Moreover, web-based versions of the three cognitive tests were developed for this study using Java with the GoogleWeb Toolkit (http://www.gwtproject.org), and were accessible from all browsers. A description of each test is given below.

#### Working memory

An adapted version of the Automated Operation Span Task (OSpan; [54]), a computerized form of the complex span task created by Turner and Engle [38], was used to gauge participants’ working memory capacity [9, 22]. Based on the Klingon Span Task implemented by Hicks et al. [22], this version consisted of using Klingon symbols instead of letters, the stimuli to be remembered in the original OSpan task. In Hicks et al.’ study, participants cheated by writing down the letter memoranda in the web-based version of the classic OSpan, motivating the change of the original stimuli. The task included a practice phase and a testing phase. In the practice phase, participants were first shown with a series of Klingon symbols on the screen, and then were asked to recall them in the order in which they had appeared after each trial (i.e., symbol recall). Next, participants were required to solve a series of simple equations (e.g., 8 * 4 + 7 = ?). Finally, subjects performed the symbol recall while also solving the math problems, as they would later do in the actual testing phase. Following the practice phase, participants were shown with the real trials, which consisted of a list of 15 sets of 3–7 randomized symbols that appeared intermingled with the equations. In sum, there were 75 symbols and 75 math problems. At the end of each set, participants were asked to remember the symbols in the sequence they had been presented. An individual time limit to answer the math problems in the real trials was calculated from the average response time plus 2.5 standard deviations taken during the math practice section. Following Unsworth et al. [54], a partial score (i.e., total number of correct symbols recalled in the correct order) was taken as the OSpan score (see [39], for a description of scoring procedures). The highest possible score was 75. The entire task took about 25 min.

#### Verbal declarative memory

To measure verbal declarative memory, the Modern Language Aptitude Test, Part 5, Paired Associates (MLAT5; [51]), was used [9, 49, 50]. In the MLAT5, participants were required to memorize artificial, pseudo-Kurdish words and their meanings in English. Participants were first asked to study 24-word association pairs for two minutes, and then complete a two-minute practice section. The list of foreign words with their respective English meanings was made available for participants as they completed the practice session. Finally, subjects were instructed to complete a timed multiple-choice test (four minutes), by selecting the English meaning of each of the 24 pseudo-Kurdish words from five options previously displayed at the memorization stage. For each correct response, one point was given, yielding a total score of 24 points. The test duration was about 8 minutes.

#### Nonverbal declarative memory

The Continuous Visual Memory Task (CVMT; [52]) served as a measure of nonverbal declarative memory [9, 49, 50]. As a visual recognition test, the CVMT is entails asking participants to first view a collection of complex abstract designs on the screen, and then to indicate whether the image they just saw was novel (“new”) in the collection, or they had seen the image before (“old”). Seven of the designs were “old” (target items), and 63 were “new” (distractors). The target items appeared seven times (49 trials), and the distractors only once (63 trials) across the test. All items were shown in a random but fixed order, each one appearing on the screen for two seconds. Following the two seconds, participants were instructed to respond to the “OLD or NEW?” prompt on the screen. In the lab-based mode, subjects used mouse click for making their choice, left for “NEW”, or right for “OLD”. In the web-based mode, they responded by pressing either the “N” key for “NEW”, or the “O” key for “OLD” on the keyboard. The CVMT took 10 min to complete. A *d*’(d-prime) score [55] was calculated for each participant. The *d*’ score was used to reduce potential response bias.

### Procedure

As previously noted, participants underwent two cognitive testing sessions, one in the lab and one on the web. In the lab-based session, with the assistance of a proctor, each subject was tested individually. After providing informed consent, participants took the three cognitive tests under investigation in fixed order: OSpan, CVMT, and MLAT5. Upon finishing the MLAT5, subjects then filled out a background questionnaire. The whole lab-based session lasted about 40 min.

Regarding the web-based session, each subject was sent an email with a unique web link with a personalized code, which once clicked, took them to an interface that hosted the web-based versions of the cognitive tests. In order to avoid multiple responses by the same participant, the link was disabled once subjects had submitted their responses in the last test (i.e., MLAT5). In the email, participants were also informed that the web-based session lasted about 40 min, and that it had to be completed within a week. On the interface, following informed consent, subjects were provided with general instructions that reflected the nature of a web-based experiment. Such instructions included completing the experiment in a quiet place without interruption, and from start to finish in one sitting. Likewise, the use of the browser’s back button, refreshing the browser page, or closing the browser window were prohibited. Importantly, participants were instructed not to take any notes at any point during the entire experiment. The web-based tests were given in the same fixed order as in the lab-based session. On average, the mean period between the first and second testing was 45.7 days (*SD* = 4.1).

## Results

All data were analyzed by means of R (version 3.3.2; [56]). Missing data was ignored (complete-case analysis). Linear regression models were built using the lm function in the lme4 library [57]. From a temporal perspective, lab scores were used to predict web scores in the linear regression models. To verify normality, model residuals were visually inspected. Reliability was assessed using Cronbach's alpha. Following Kane et al. [58], for the lab-based working memory test (OSpan-Lab-based), reliability was assessed by calculating the proportion of correctly recalled Klingon symbols per each of the 15 trials in the test (e.g., one out of four symbols correctly recalled corresponded to a proportion of .25). For the web-based working memory test (OSpan-Web-based), however, internal consistency is not reported, since it was not technically possible to perform a detailed item-based analysis. Descriptive statistics are presented first, followed by correlations, internal consistency estimates (Cronbach's alpha), and the results of linear regression analyses.

### Descriptive statistics

Table 1 presents the descriptive statistics for participants’ performance on cognitive tests in both testing settings.

### Correlations

**Fig 1:**
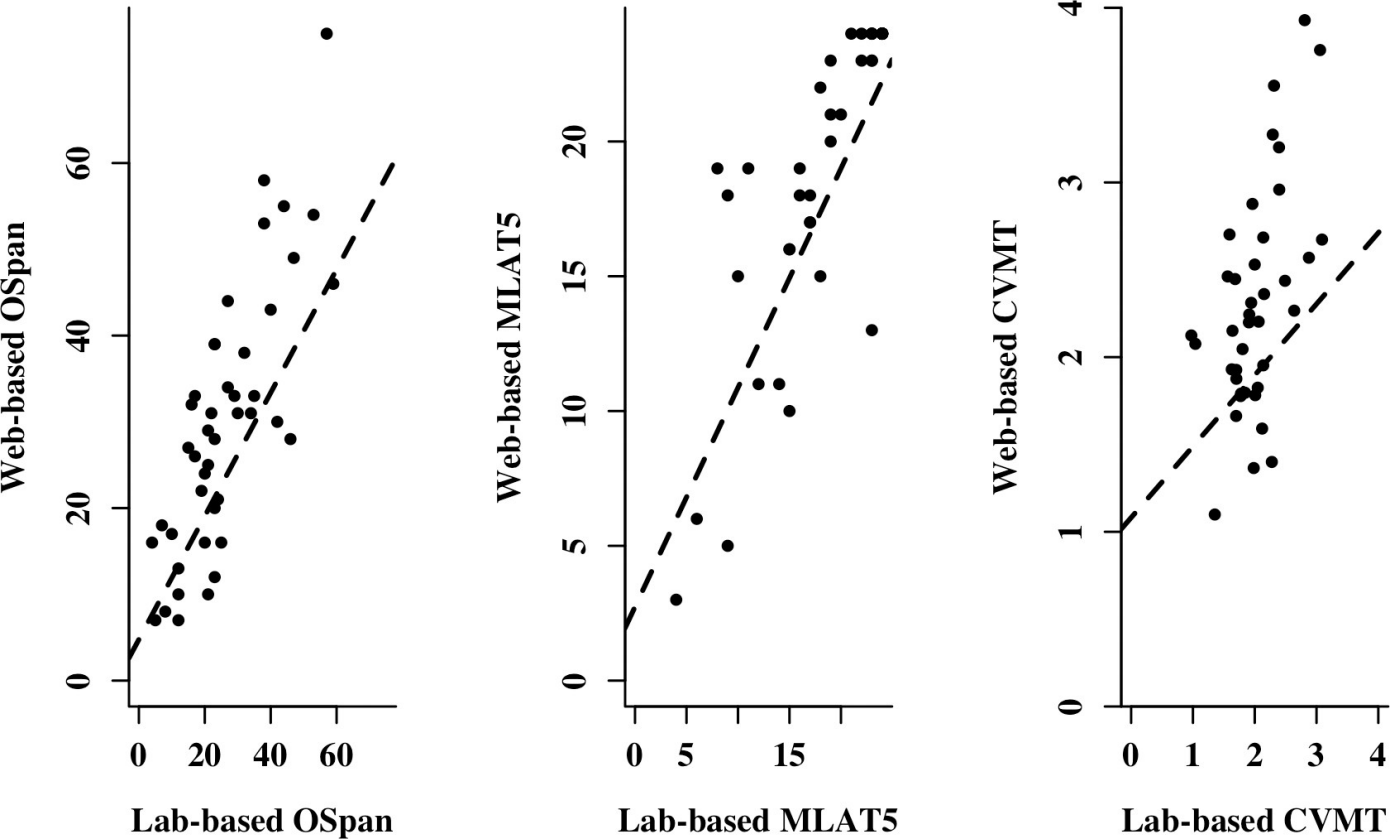
Scatterplots of the correlation of each pair of lab-based and web-based versions of individual difference measures. OSpan = Automated Operation Span Task; Verbal declarative memory test: MLAT5 = Modern Language Aptitude Test, Part 5; Nonverbal declarative memory test: CVMT = Continuous Visual Memory Task.

Table 2 and Fig 1 show the correlations between/among the different versions of the individual difference tests.

**Table 2:** Correlations between lab-based and web-based scores for individual difference tests.

Test	OSpan Lab-based	OSpan Web-based	MLAT5 Lab-based	MLAT5 Web-based	CVMT Lab-based
OSpan Web-based	.80				
MLAT5 Lab-based	.40	.51			
MLAT5 Web-based	.32	.40	.82		
CVMT Lab-based	.19	.31	.42	.23	
CVMT Web-based	.21	.30	.21	.19	.55

Note: OSpan = Automated Operation Span Task; Verbal declarative memory test: MLAT5 = Modern Language Aptitude Test, Part 5; Nonverbal declarative memory test: CVMT = Continuous Visual Memory Task.

### Reliability

Table 3 presents Cronbach's alpha values of individual test versions.

**Table 3:** Cronbach’s alphas for cognitive test versions.

Test	Cronbach’s alpha
OSpan Lab-based	.86
MLAT5 Lab-based	.77
MLAT5 Web-based	.93
CVMT Lab-based	.63
CVMT Web-based	.67

Note: OSpan = Automated Operation Span Task; Verbal declarative memory test: MLAT5 = Modern Language Aptitude Test, Part 5; Nonverbal declarative memory test: CVMT = Continuous Visual Memory Task.

### Regression analysis

The results of the regression analyses are displayed in Table 4. For the working memory test (OSpan), the unstandardized coefficient was .89 (*β* = .77, *SE* = 0.10, *p*<.001). For the verbal declarative memory test (MLAT5), the unstandardized coefficient was .83 (*β* = .78, *SE* = 0.09, *p*<.001). And for the nonverbal declarative memory test (CVMT), the unstandardized coefficient was .74 (*β* = .54, *SE* = 0.19, *p*<.001). Overall, the results indicated that the lab-based and web-based scores are substantially related.

**Table 4:** Regression for comparison of lab-based and web-based scores.

Test	Unstandardized coefficient^a^	*SE*	*p*
OSpan	0.89 (.77)	0.10	< .001
MLAT5	0.83 (.78)	0.09	< .001
CVMT	0.74 (.54)	0.19	< .001

Note: OSpan = Automated Operation Span Task; Verbal declarative memory test: MLAT5 = Modern Language Aptitude Test, Part 5; Nonverbal declarative memory test: CVMT = Continuous Visual Memory Task.

^a^The standardized coefficient (*β*) in parentheses.

## Discussion

Studies on individual differences in language learning frequently assess the working memory and declarative memory capacities of their participants in order to determine the effect of these cognitive variables on learning outcomes. Most of this research, however, is conducted in lab-based settings, which often implies relatively small sample size and a restricted population sample. Both of these methodological challenges can be addressed by means of remote testing via the web. In the present study, we compared lab-based and web-based individual difference measures in order to validate web-based tests for future research. The type of comparison contributes significantly to ongoing efforts to improve the methodological robustness of current second language research, for example [12]. If web-based testing can be shown to yield comparable results to lab-based testing, researchers will be able to reach more participants for their studies, which, in turn, can help alleviate some of the current concerns in lab-based research (e.g., low statistical power, non-representative population samples, and small sample sizes). In addition, demonstrating the equivalence of lab-based and web-based measures of the same individual difference constructs is essential for the comparability of results across studies. Crucially, establishing measurement equivalence between lab-based and web-based versions will also provide assurance that the tests are measuring cognitive constructs the same way regardless of administration mode [17, 59].

Findings showed that the scores in the lab-based and web-based versions of three cognitive tests (MLAT5, CVMT, OSpan) were equivalent concerning differences in performance, which were constant in the two versions, suggesting that participants who had relatively high values in one task also had relatively high values in the second, or the other way around. However, the strength of the relationship was a function of the kind of test. More specifically, in both the working memory test (OSpan) and the verbal declarative memory test (MLAT5), the scores were more strongly correlated (*β* = .77 and *β* = .78, respectively); for the nonverbal declarative test (CVMT), equivalence appears to be weaker (*β* = .54).Overall, the correlations reported here between lab-based and web-based scores are consistent with the assumption that both versions seem to likely measure the same cognitive construct, at least for the working memory test (OSpan) and the verbal declarative memory test (MLAT5), and, to a lesser extent, for the nonverbal declarative test (CVMT).

A potential explanation for lesser equivalence in the versions of the nonverbal declarative test (CVMT) could be due to the different manner in which the responses to the visual stimuli were entered in the two testing modes. It will be recalled that in the lab-based version participants used left (“NEW”) or right (“OLD”) mouse clicking to provide a response, whereas in the web-based version, they used the keyboard (“N” and “O” keys). This modification made to the web-based version was motivated by technical reasons, specifically, the browser window may not register the participants’ response if the cursor is not over a certain area on the page, which in turn may cause problems of missing data. Previous research has found that participants in web-based research are particularly prone to err when using the keyboard to input their responses [60], which in this case might have affected the results of the comparison between lab-based and web-based versions of CVMT. Future research comparing performance between the lab and web-versions may benefit from collecting data through touch input instead, as this might help overcome potential technical difficulties caused by using mouse clicking for web-based data.

Some limitations of the study and the findings presented here should be considered. One of the limitations was the small sample size. As mentioned earlier, logistic constrains due to the availability of time and funding prevented the researchers from testing more participants for this study. In addition, the fact that some participants (14%) dropped out before completing any of the web-based measures in the second part of the experiment, which is typical in web-based research [17], also contributed to the reduction of the data available for the comparison between lab and web-based testing in the present investigation. Therefore, our findings should be replicated in a larger study. A second limitation was that test-retest reliability was not examined here, given that the main aim of this study was to establish valid online versions of known individual difference measures. Future research should assess test-retest reliability, as it is as an interesting endeavor for studying individual difference measures in future work. Finally, and as indicated above, a third limitation concerned technical issues that affected data collection, as some participants used the wrong keys on the keyboard to submit their responses to the web-based version of the CVMT, rendering the data from some of the participants impossible to use for the comparison; furthermore, data from one subject was missing in the Web-based MLAT, which may have been due to technical issues at the participant’s end (e.g., not following the general instructions given, such as refreshing or closing the browser page [see Procedure]; or Internet disconnection). In this sense, Reips and Krantz [61] (see also [17]) caution researchers that one of the potential disadvantages of Internet-driven testing is the technical variability characteristic of web-based research (e.g., different browsers and Internet connections), which, in turn, may affect data collection.

## Conclusion

This study aimed to establish the validity of using web-based versions of established offline tasks. As such, the study has provided evidence that it is possible to measure individual differences in cognitive abilities on the web and obtain similar performance as in the lab. The lab-based and web-based versions of the three cognitive tests are comparable or equivalent. However, given that they do not perfectly correlate, we recommend using one of the two modes within one study and not comparing individual scores from one mode with scores from the other. Moreover, the extent to which the measures are equivalent varies according to the test. In this sense, we are confident that the two versions for the working memory test (OSpan) and the verbal declarative memory (MLAT5) are likely to measure the same construct, whereas the correlation between the nonverbal declarative test (CVMT) versions was less pronounced. Our research has shown that collecting experimentally controlled data on cognitive individual differences typically used in the area of L2 research in the Internet is feasible and comparable to lab-based collection. Consequently, some of these web-based versions could very well be incorporated, for example, in future web-based intervention studies on second language learning, thereby contributing to the scaling up of data collection in the field [62–64].

## Supporting information

S1 Dataset Dataset behind analyses in ‘Measuring individual differences in cognitive abilities in the lab and on the web’. (CSV)
